# Ratings of Perceived Exertion Misclassify Intensities for Sedentary Older Adults During Graded Cycling Test: Effect of Supramaximal High-Intensity Interval Training

**DOI:** 10.3389/fphys.2018.01505

**Published:** 2018-10-25

**Authors:** Georges Jabbour, Lina Majed

**Affiliations:** Sport Science Program, College of Arts and Sciences, Qatar University, Doha, Qatar

**Keywords:** older individuals, rating of perceived exertion, ventilatory threshold, sedentary, supramaximal cycling exercise

## Abstract

The present study aims (1) to evaluate ratings of perceived exertion (RPE) and corresponding intensities during a maximal graded cycling test and (2) to determine the effects of 6 weeks of supramaximal cycling exercise (SCE) intervention on RPE and associated physiological factors in young and older sedentary groups. Two healthy groups of 17 young adults [average (SD) age: 26.2 (2.4) year] and 13 older adults [average (SD) age: 54.5 (2.3) year] completed a 6-week SCE intervention on an ergocycle. Physiological values and RPE were collected across stages corresponding to ventilator thresholds 1 (VT1) and 2 (VT2) of the graded cycling test and 10 min following the end of test and during the six bouts of SCE. The relative intensity for both VT1 and VT2 were also objectively calculated based on the percent of maximal heart rate %HRmax and peak oxygen consumption %$\dot{V}$O_2_peak.

Before SCE intervention, RPE values were significantly higher for the older group compared to younger at VT1 [*p* < 0.01] and VT2 [*p* < 0.01], although both groups were working at similar relative intensities (%$\dot{V}$O_2_). After 6 weeks of SCE, the older group’s perceived effort values were normalized to the actual estimated ones and were similar to those observed in younger individuals. The intervention elicited physiological changes at rest and submaximal intensities, while no improvements were noted for both groups in aerobic fitness (i.e., $\dot{V}$O_2_peak). For both groups, RPE decreases with SCE at 10 min following graded test correlated significantly to $\dot{V}$O_2_ (*r* = 0.61, *p* < 0.01). Our study revealed that the initial over-estimation of the exertion levels found for the older sedentary group at the tested submaximal intensities was no longer present after 6 weeks of SCE training, therefore matching RPE values of the young group and those estimated by %HRmax and %$\dot{V}$O_2_peak methods. Therefore, combining the RPE method with other commonly used methods of estimating exercise intensity is highly recommended for sedentary older adults to suitably monitor the exercise intensity.

## Introduction

As the world’s population is aging with most countries experiencing an increase in the portion of older adults, there is a growing need to prevent morbidity and improve the quality of life with advancing age. Exercise is an effective and valuable alternative to provide health benefits and prevent from multiple deleterious effects of aging (Hale and Marshall, 2017; Reid and Foster, 2017). Many recommendations are available today to guide exercise prescription for health and disease. The most commonly used guideline refers to 30 min of moderate-intensity aerobic (endurance) physical activity on 5 days each week or 20 min of vigorous-intensity aerobic physical activity on 3 days each week to promote or maintain health (Haskell et al., 2007). However, several barriers to exercise have revealed the importance of adherence and an appropriate administration of the prescribed exercise (e.g., intensity).

A little more than a decade ago, exercise scientists have started exploring the benefits of high-intensity interval training (HIIT) characterized by brief, intermittent bouts of vigorous-intensity exercise, interspersed by periods of rest or active recovery (Gibala et al., 2012). This relatively new method of training is showing comparable outcomes to those of a traditional moderate-intensity longer-duration exercise (Gibala et al., 2009). HIIT has proven to be an efficient alternative to promote aerobic fitness as well as several health parameters over brief periods of time (Gillen and Gibala, 2013) or even with a single session per week (Matsuo et al., 2014). Despite the potential benefits of HIIT, some studies reported that such high-intensity model of training induced discomfort, which is deterrent to long-term adherence (Ekkekakis et al., 2011; Foster et al., 2015). At the contrary, other studies showed that HIIT represents an attractive training strategy compared with continuous aerobic exercise (Jung et al., 2015) and might be achieved with a lower rate of perceived effort (Bangsbo et al., 2015). Perhaps the most salient characteristic of HIIT remains its time-efficiency. Chao et al. (2000) have reported that older adults perceive exercise participation to be overly time-consuming, which represents a critical barrier to any exercise program adherence.

It has been recently acknowledged that most studies on HIIT were performed on young adults and that more focus should be given to older participants in order to better understand the effects of HIIT on that portion of the population (Weston et al., 2014). HIIT remains a multimodal training with a broad potential of application. Each HIIT form can differ significantly from others depending on the duration and intensity of each interval, the number of intervals performed, and the duration of recovery (relative to the effort time) between bouts of effort. Different HIIT protocols have shown varying physiological and psychological adaptive responses (Ross and Leveritt, 2001). To date, the most common exercise that has been used in obesity management for instance, is the Wingate test. The latter consisting of 30 s of all-out sprinting induced many beneficial effects in overweight individuals (Whyte et al., 2010), however, this protocol remains extremely difficult, and participants have to tolerate some substantial discomfort. More recently, Jabbour et al. (2016) reported that very brief (i.e., 6 s) high-intensity exercise in the form of sprinting induced substantial improvements in both performance and health-related outcomes in similar obese participants. In addition, this form of supramaximal and very brief exercise is motivating and tolerated well by participants, which is reflected by the excellent compliance to the intervention. These results highlight the potential of this exercise model to provide an alternative exercise intervention for the improvement of health among populations with or at risk of health problems. Moreover, monitoring adequately exercise load to determine whether an individual is appropriately targeting the prescribed exercise training can promote effectiveness of the intervention especially among a very susceptible population such as sedentary older individuals.

Today, there is a number of potential indicators used to increase our understanding of the exercise load and its effect on the individual. The rating of perceived exertion (RPE) is one of the most popular and reliable tools that provides an understanding of physiological stress during exercise as well as retrospective information regarding perceived effort during exercise (Halson, 2014). Perceived exertion is a psycho-physiological marker of intensity resulting from a complex integration of subjective feelings of effort, strain, discomfort, and/or fatigue experienced during exercise (Robertson and Noble, 1997). To optimize exercise-derived health benefits in older individuals it seems necessary that the prescribed exercise be tailored and individualized according to reported subjective measures of intensity. For healthy sedentary and special populations (e.g., persons with cardiovascular or metabolic diseases) HIIT has been used (Whyte et al., 2010; Gillen and Gibala, 2013; Jabbour et al., 2016) and often prescribed based on heart rate and oxygen uptake responses during incremental exercise testing (Coquart et al., 2008; Akbarpour, 2013; Biddle and Batterham, 2015). While these methods require sophisticated equipment and laboratory spaces, the use of RPE presents an important advantage for prescribing and self-regulating the effort in a real-exercise setting. In healthy middle-aged and elderly individuals, RPE was shown not to be impaired by aging and to be associated with heart rate to control exercise intensity (Sidney and Shephard, 1977; Aminoff et al., 1996). Interestingly, Grange et al. (2004) did not find a significant relationship between RPE and HR in physically deconditioned older persons (75.2 years). However, following 6 weeks of arm training, a significant HR-RPE relationship was found in most of the subjects. For non-elderly individuals other variables were also combined with RPE (e.g., oxygen uptake, ventilator threshold) to assess and regulate the intensity of exercise. While much is known about RPE responses and related factors in children and adults, little is known about the perceptual responses to exercise (i.e., RPE) or associated factors in older sedentary members of the population. Whether RPE can be used appropriately and accurately in such groups needs to be further explored given their different response and tolerance to exercise, in order to be more widely used in the health and clinical sectors.

With the growing interest of promoting exercise prescription among elderly population, optimizing the health benefits as well the adherence to exercise programs remains the major concern. Despite the promising HIIT and sprint interval training (SIT) results, no study has yet examined the changes in perceptual responses (i.e., RPE) following supramaximal HIIT (SIT) and their relationship with physiological outcomes in a sedentary population of young and older adults. Assuming that the RPE assesses adequately the exercise intensity for older and younger individuals, the present study aims to compare RPE and associated factors in young and older sedentary individuals before and after 6 weeks of supramaximal HIIT on ergocycle, more commonly known as supramaximal cycling exercise (SCE). For the purpose of the current work, RPE and physiological responses were evaluated at different relative submaximal and maximal intensities and after 10 min of recovery during two maximal graded cycling tests performed before and after the SCE program. RPE and physiological responses were also examined immediately at the completion of two six bouts of supramaximal cycling tests realized at the start and end of the SCE program. We hypothesized that RPE presents an interesting tool to estimate suitably the exercise intensity for older individuals and SCE intervention improves RPE concomitantly with the improvement of physiological and fitness variables as reported for adults.

## Materials and Methods

### Participants

Seventeen healthy young adults [12 female and 5 male; average (SD) age = 26.2 (2.4) years old] and thirteen healthy old adults [8 female and 5 male; mean (SD) age = 54.5 (2.3) years old] volunteered for the study. Participants were classified according to growth stages: young (18–40 years) and older adults (41–71 years). The sample consisted of university students and staff who were recruited through posted announcements. Physical characteristics of both groups are presented in Table 1. The inclusion criteria for participation were as follows: (i) be sedentary [<60 min.week^-1^ of structured exercise, as assessed by the International Physical Activity Questionnaire (Craig et al., 2003)], (ii) not be taking part in any systematic exercise training at the time of study or during the 6 months that preceded the study, (iii) no history of orthopedic, neurological, cardiovascular or other chronic disease, (iv) no history of drug consumption or (v) smoking. The procedures were approved by the University’s Human Research Ethics Committee (UHRC), and performed in accordance with the Helsinki Declaration of 1975, as revised in 2008. Informed consent was obtained from all participants prior to start of the study.

**Table 1:** Results on anthropometric data before (pre-intervention) and after (post-intervention) the supramaximal cycling exercise (SCE) intervention for both young and older groups.

	Pre-intervention		Post-intervention		Interaction Effect	
			
	Young	Older	Young	Older	*F*	*p*
Height *(m)*	1.69 (1.1)	1.68 (1.3)	1.71 (1.1)	1.68 (1.3)	1.6	0.33
Body mass *(kg)*	82.3 (3.8)	90.1 (2.8)^a^	82.2 (1.1)	87.1 (3.3)^ab^	11.6	<0.01
BMI *(kg.m^-*2*^)*	28.6 (1.1)	31.9 (1.1)^a^	28.7 (1.4)	30.5 (1.5)^ab^	12.7	<0.01
FM *(kg)*	27.1 (1.1)	33.8 (2.6)	26.4 (1.7)	31.8 (2.9)^ab^	20.1	<0.01
FFM *(kg)*	56.1 (2.1)	56.2 (1.1)	57.1 (2.1)	56.1 (1.3)	0.4	0.63


*Values are mean ± SE (standard error). BM, body mass; BMI, body mass index; FM, fat mass; FFM, fat free mass. ^*a*^Significant difference between groups (*p* < 0.01). ^*b*^Significant difference from pre-intervention values within an age group (*p* < 0.01).*

### Protocol

The protocol consisted of two testing sessions performed before (pre-intervention) and after (post-intervention) a 6-week supramaximal HIIT program on a cycle ergometer, referred to here as supramaximal cycle exercise (SCE). Pre-intervention testing sessions determined baseline levels of key variables, while the post-intervention testing sessions examined changes in key values inferred by the SCE program. Each of the pre- and post-intervention testing sessions followed the same procedures and were conducted on two different days (Day 1 and Day 2) separated by a minimum of 48 h, and took place in the morning of each day (∼ 8.30am) after an overnight fast. For the testing sessions, non-menopausal female participants were in the follicular phase of their menstrual cycle. During the 6 weeks of intervention all participants completed all of the training sessions (thus adherence was 100%) and no other difficulties or incidents were encountered. Two supramaximal cycling tests were further conducted at the first and last SCE sessions as part of the training. The current protocol has been developed and previously used by our team (Jabbour et al., 2018). Before starting the experiment, participants were thoroughly familiarized with the equipment and testing procedures and were instructed on how to indicate the RPE (6–20 Borg scale, Borg, 1970) when requested by the experimenter. The range of sensations that correspond to categories of effort within the scale were clearly explained to each participant.

#### Pre- and Post-intervention Testing Sessions

At baseline on Day 1 and after assessing body composition, participants performed an incremental maximal test on a cycle ergometer (Ergomedic 839E, Monark, Sweden) with continuous measurement of pulmonary gas exchange using a breath-by-breath automated metabolic system (Ergocard MEDI-SOFT, Sorinnes, Belgium) to determine peak oxygen consumption ($\dot{V}$O_2_peak). Calibrations were performed prior to each test using standard gasses of known oxygen and carbon dioxide concentrations as well as a calibration syringe for air flow. Before beginning the test, participants remained seated for 5 min on the bicycle ergometer in the same position as that used for exercise to measure resting values. The test began at an initial power of 25 watts and increments of 25 watts followed every 5 min until exhaustion. During the test, participants were instructed to pedal at a rate of 50–70 revolutions per minute. The test was terminated when the participants requested to stop the exercise or could no longer maintain the required pedaling rate (<40 revolutions per minute). A recovery phase of 5 min at 25 watts followed the test (Jabbour et al., 2018).

After a 48-h rest on Day 2 and following a 10-min warm-up, participants performed a Force-Velocity test on a cycle ergometer using a technique adapted from the study of Vandewalle et al. (1988). This test consists of a succession of supramaximal bouts of approximately 6 s, with flywheel resistance increasing by 1 kg after each bout until the subject is unable to perform the test. A period of passive recovery (5 min) was allowed between successive bouts. The peak velocity for each bout was recorded, and the power output was calculated by multiplying the load with the speed. The optimal load corresponded to the load at which maximal power (Pmax) was achieved. This load was then used for the SCE protocol that followed. The Force-Velocity test was also performed every 2 weeks to adjust the individual power level used during SCE.

#### SCE Intervention and Supramaximal Tests

Once participants completed the preliminary testing, a total of 18 SCE training sessions was prescribed over a period of 6 weeks (three sessions per week). The same training protocol has been previously developed and tested by our laboratory (Jabbour and Iancu, 2015; Jabbour et al., 2015, 2018). Each of the prescribed sessions began with a 5-min warm-up consisting of continuous cycling at moderate intensity corresponding to 40–50% of each participant’s maximal heart rate (HRmax), and was followed by 6 repetitions of SCE intervals with 2 min of passive recovery between each repetition. Each SCE repetition lasted 6 s, and participants were asked to pedal at maximal velocity against the resistance that was determined on Day 2. Heart rate values were monitored during all training sessions using a heart rate monitor (Polar, Kempele, Finland). The total duration of each session was approximately 16–18 min. During the training sessions, the velocity (in revolutions per minute) was recorded for each second of the entire round to ensure that participants pedaled at their maximal capacity. Indeed, values varied among individual in range of ∼160–200 rpm.

Additional testing was also completed during the first and the last (18th) SCE training sessions. Indeed, during these two testing sessions, participants were asked to perform one of their regular training sessions, while power output and heart rate values obtained for the 6 SCE repetitions were recorded.

### Data Analysis

In the present work, the training adherence of the participants was calculated as the percentage of the actual number of training sessions completed in compliance with the targeted intensity and duration, relative to the total number of training sessions prescribed.

#### Anthropometric Data

Body mass, fat-free mass and fat mass were assessed using bio-impedance scale (Bodystat1500, Isle of Man, British Isles). Height was determined to the nearest 0.5 cm with a measuring tape affixed to the wall. Body mass index (BMI) was calculated as the ratio of mass (kg) to height squared (m^2^).

#### Pre- and Post-intervention Testing Data

For $\dot{V}$O_2_peak tests, ventilatory and gas exchange data were collected on a breath-to-breath basis along with continuous heart rate (HR, beats.min^-1^) measurements. Data on minute ventilation $\dot{V}$_E_, (L.min^-1^), oxygen consumption ($\dot{V}$O_2_, mL.min^-1^), carbon dioxide production ($\dot{V}$CO_2_, mL.min^-1^) and respiratory exchange ratio (RER) were determined at each increment level as the average of the last 20 s where a steady-state in values was reached. Systolic (SBP) and diastolic (DBP) blood pressure (mmHg) were measured at the left arm using the auscultatory method with a stethoscope and sphygmomanometers (Vaquez-Laubry, Spengler, Issoudun, France) and respectively averaged over three recordings. Ratings of perceived exertion (RPE, Borg, 1970) were collected at the end of each increment level by asking participants to raise their arm to indicate the RPE value as the experimenter read up the Borg scale. The latter ranged from 6 to 20 with 7 indicating that the effort is *very very light*, and 19 indicating that the effort is *very very hard*.

For the purpose of the analysis, $\dot{V}$O_2_, HR, SBP, DBP, and RPE were reported at 5 different moments of the pre- and post-intervention’s maximal incremental cycling tests: (i) prior to start at rest (except for RPE), (ii) at the first and (iii) second ventilatory thresholds (VT1 and VT2), (iv) at the maximal workload ($\dot{V}$O_2_peak) and (v) 10 min after the completion of the test (recovery). Peak oxygen consumption ($\dot{V}$O_2_peak) was determined using the following criteria: (1) a peak or plateau in oxygen uptake values despite an increase in exercise intensity, (2) respiratory exchange ratio ≥ 1.1, (3) peak heart rate ± 10 beats.min^-1^ of the predicted maximal heart rate (220 - age) and (4) voluntary exhaustion indicated by an RPE > 17 (Spiro, 1977; Howley et al., 1995). In the present study, $\dot{V}$O_2_peak was determined as $\dot{V}$O_2_ mean of the final 20 s of each stage, and the $\dot{V}$O_2_peak was assumed as the highest $\dot{V}$O_2_mean reached in incremental maximal test (Malta et al., 2018).

Ventilatory thresholds were determined using established criteria as per Wasserman et al. (1999) and used to classify the intensity of aerobic exercise. Briefly, VT1 corresponds to the break point in the plot of $\dot{V}$CO_2_ as a function of $\dot{V}$O_2_. At that point, $\dot{V}$_E_/$\dot{V}$O_2_ increases without an increase in $\dot{V}$_E_/$\dot{V}$CO_2_. VT2 was located between VT1 and $\dot{V}$O_2_peak when $\dot{V}$_E_/$\dot{V}$O_2_ begins to increase and $\dot{V}$_E_/$\dot{V}$CO_2_ continues to increase. VT1 and VT2 were determined independently by three experienced investigators. At these two stages, we determined the relative intensity corresponding to the percentage of maximal heart rate (%HRmax) and to the percentage of peak oxygen consumption (%$\dot{V}$O_2_peak) (Garber et al., 2011).

As for the supramaximal testing, power output (P, W.kg^-1^), HR, %HRmax and RPE were collected for each of the six repetitions of the first and last SCE training sessions. RPE was obtained immediately after the end of each 6-s interval.

### Statistical Analyses

Before the analysis, all datasets were tested for normality using the Kolmogorov–Smirnov test. ANOVAs with 2 × 2 repeated measures [Intervention (pre- and post-HIIT intervention) × Group (young and older)] were performed on all variables (anthropometric, physiological, RPE and power output) collected for the 5 key moments of the incremental maximal test as well as during each of the 6 repetitions of the supramaximal testing. When a significant interaction effect was found, the analysis was completed with Bonferroni’s *post hoc* for pairwise comparisons. Pearson correlations were used to assess the association between RPE and anthropometric, physiological and fitness variables. The analyses were performed using IBM SPSS Statistics 19 software (IBM SPSS Statistics for Windows, Version 24.0. Armonk, NY, United States: IBM Corp.). A value of *p* < 0.05 was considered statistically significant for all tests.

## Results

Of the 30 eligible participants, none withdrew from the intervention. Accordingly, the data provided from 100% of the total sample was used for subsequent analyses.

### Anthropometric Variables

ANOVA’s results on anthropometric variables are presented in Table 1. At the pre-intervention test, an initial significant difference in body mass and BMI values was detected between the young and older groups and maintained at post-intervention test. The (intervention × group) interaction effect reported on body mass, BMI and FM was explained by the significant decrease in values following the intervention only for the older group, while no significant changes were incurred by the SCE program for the young group.

### Incremental Maximal Tests

During the maximal incremental cycling test, VT1 and VT2 were attained by both groups at similar relative intensities that did not vary significantly with the 6-week SCE intervention (Table 2).

**Table 2:** Results on relative intensities reached at the first and second ventilatory thresholds (VT1 and VT2) before (pre-intervention) and after (post-intervention) the supramaximal cycling exercise (SCE) intervention for both young and older groups.

		Pre-intervention		Post-intervention		Interaction effect	
				
		Young	Older	Young	Older	*F*	*p*
VT1	%HRmax	54 (3)	58 (4)	54 (4)	54 (1)	3.1	0.28
	%$\dot{V}$O_2_peak	51 (4)	49 (9)	48 (3)	49 (5)	2.8	0.27
VT2	%HRmax	80 (2)	80 (1)	77 (4)	75 (8)	3.2	0.29
	%$\dot{V}$O_2_peak	69 (3)	73 (4)	62 (3)	64 (7)	2.4	0.25


*Values are mean ± SE (standard error). VT1 and VT2, ventilatory thresholds 1 and 2; %HRmax, percentage of maximal heart rate; %$\dot{V}$O_*2*_peak, percentage of peak oxygen consumption. ^*a*^Significant difference between groups (*p* < 0.05). ^*b*^Significant difference from pre-intervention values within an age group (*p* < 0.05).*

#### Physiological Variables

ANOVAs’ results on physiological variables are presented in Table 3. In brief, at the pre-intervention phase and as compared to the young group, the older group had significantly higher values for $\dot{V}$O_2_, SBP and DBP mainly at rest and during VT2, while no other differences were noted at VT1, maximal workload (to the exception of SBP) or during recovery as compared to the young group. The SCE intervention improved significantly the $\dot{V}$O_2_ and SBP values for both groups at rest, VT1, VT2 and during recovery, while no intervention-related changes were found for physiological variables at the maximal workload. Furthermore, the older group improved their DBP after the SCE training to reach similar resting values as those of the younger group. The intervention also resulted in both groups improving their recovery values for HR. Moreover, the initial between-group differences in $\dot{V}$O_2_ and SBP at VT2 were no longer significant at post-intervention, while significant differences in groups were still seen for $\dot{V}$O_2_ at rest and for SBP at rest and at the maximal workload following the intervention.

**Table 3:** ANOVA results for the intervention × group interaction effects on physiological and perceptual data analyzed at rest, during the first and second ventilator thresholds (VT1 and VT2), at maximal workload and 10 min after the end of the test (recovery).

	Pre-intervention		Post-intervention		Interaction effects	
			
	Young	Older	Young	Older	*F*	*p*
***Rest***						
$\dot{V}$ O_2_ *(mL.min^-*1*^)*	616.2 (21)	730.4 (15)^a^	580.2 (18)^b^	610.6 (16)^ab^	11.8	<0.01
*HR (beats.min^-*1*^)*	75 (8)	76 (8)	76 (5)	78 (6)	1.18	0.56
SBP*(mmHg)*	113 (9)	116 (12)	107 (6)^b^	110 (11)^ab^	7.9	<0.01
DBP*(mmHg)*	74 (8)	82 (10)^a^	72 (7)	75 (3) ^b^	14.3	<0.01
***VT1***						
$\dot{V}$ O_2_ *(mL.min^-*1*^)*	1149.4 (55) (63)	1168.7 (70)	1045.6 (63)^b^	1140.6 (60)^b^	21.2	<0.01
HR *(beats.min^-*1*^)*	101 (4)	104 (3)	100 (3)	102 (3)	1.1	0.23
SBP*(mmHg)*	171 (9)	175 (4)	150 (10)^b^	155 (7)^b^	9.8	<0.01
DBP*(mmHg)*	78 (6)	84 (6)	76 (6)	79 (3)	3.1	0.53
RPE	11 (0.3)	17 (0.3)^a^	11 (0.1)	11 (0.3)^ab^	12.8	<0.01
***VT2***						
$\dot{V}$ O_2_ *(mL.min^-*1*^)*	1540.3 (91)	1717.5 (114)^a^	1434.3 (95)^b^	1490.7 (136)^b^	11.2	<0.01
HR *(beats.min^-1^)*	151 (4)	135 (6)	145 (4)	124 (5)	1.1	0.23
SBP*(mmHg)*	175 (16)	185 (18)^a^	169 (13)^b^	173 (4)^b^	9.2	<0.01
DBP*(mmHg)*	82 (7)	84 (7)	81 (8)	83 (8)	2.9	0.66
RPE	14 (0.3)	19 (0.6)^a^	14 (0.2)	14 (0.3)^b^	11.2	<0.01
***Maximal Workload***						
$\dot{V}$ O_2_peak *(mL.min^-*1*^)*	2221.7 (485)	2338 (511)	2278(580)	2298 (380)	3.1	0.63
HRmax *(beats.min^-*1*^)*	187 (3)	177 (4)	188 (4)	179 (5)	5.1	0.33
SBP *(mmHg)*	189 (14)	170 (11)^a^	186 (9)	169 (15)^a^	10.01	<0.01
DBP *(mmHg)*	87 (3)	89 (4)	86 (8)	88 (6)	1.8	0.11
RPE	20 (0.3)	20 (0.3)	20 (0.2)	20 (0.3)	1.8	0.11
***Recovery***						
$\dot{V}$ O_2_ *(mL.min^-*1*^)*	1749.6 (63)	1768.8 (70)	1445.4 (63)^b^	1440.5 (60)^b^	11.01	<0.01
HR*(beats.min^*-1*^)*	150 (4)	155 (3)	134 (3)^b^	135 (3)^b^	21.01	<0.01
SBP*(mmHg)*	161 (9)	162 (4)	151 (10)^b^	153 (7)^b^	11.2	<0.01
DBP*(mmHg)*	79 (6)	81 (6)	78 (8)	81 (3)	3.8	0.63
RPE	17 (0.3)	17 (0.3)	12 (0.2)^b^	12 (0.3)^b^	19.8	<0.01


*Values are mean ± SE (standard error). HR, heart rate; $\dot{V}$ O_*2*_, oxygen consumption, $\dot{V}$ O_*2*_peak, peak oxygen consumption, SBP: systolic blood pressure, DBP: diastolic blood pressure, RPE: rating of perceived exertion. ^*a*^Significant difference between groups (*p* < 0.01). ^*b*^Significant difference from pre-intervention values within an age group (*p* < 0.01).*

#### Rating of Perceived Exertion

Table 3 presents ANOVAs’ results on RPE. At the pre-intervention phase, the older group perceived a significantly higher effort at the relative submaximal intensities VT1 and VT2 as compared to the young group. Indeed, at VT1 the older group judged the effort as being *very hard* compared to a *fairly light* rating for the young group, knowing that the actual relative intensity did not differ between groups (49–51%$\dot{V}$O_2_peak). This was also the case at VT2, where the older group judged the effort as being *very very hard* compared to a *somewhat hard to hard* for the young group, while the actual relative intensity was similar between groups (80%$\dot{V}$O_2_peak). The intervention resulted in a significant decrease in the RPE values for both groups at the recovery phase (10-min post-exercise) from *very hard* to *somewhat hard to hard*. RPE only decreased significantly for the older group at both relative submaximal intensities thus bringing their perception of effort to the same levels as those seen in the young group (VT1: *fairly light*; VT2: *somewhat hard to hard*). At $\dot{V}$O_2_peak, RPE values reached their maximal level for both groups.

At 10 min post-graded cycling test (recovery), RPE decreases correlated positively with $\dot{V}$O_2_ for both groups (*r* = 0.61; *p* < 0.01). However, no significant associations were found between RPE and both post-intervention BM and HR decreases.

### Supramaximal Tests

After the SCE intervention, no significant changes were obtained for RPE values among groups compared with those observed at baseline. The RPE values corresponded to *fairly light* for both groups. During SCE, the relative intensity was 54% of maximal heart rate for both groups at baseline and at post-intervention (Table 4). In this study, the RPE obtained for both groups during the six bouts of SCT at baseline and at post-intervention correlated significantly with HR values (*r* = 0.61; *p* < 0.01). No significant correlation was obtained between RPE and muscle power increases observed for both groups at post-intervention (*r* = 0.002; *p* = 1.1).

**Table 4:** Results on measured variables during the 6 repeated supramaximal cycling exercises at the first (pre-intervention) and the last (post-intervention) HIIT session for both young and older groups.

	Pre-intervention		Post-intervention		Interaction effect	
			
	Young	Older	Young	Older	*F*	*p*
***1st repetition***						
P1 *(W.kg^*-1*^)*	6.2 (0.3)	6.2 (0.5)	8.5 (1)^b^	7.5 (0.5) ^ab^	14.01	< 0.01
HR *(beats.min^*-1*^)*	104 (3)	102 (2)	104 (3)	102 (2)	4.1	0.23
%HRmax	54	55	54	54	2.1	0.23
RPE	11 (0.2)	11 (0.6)	11 (0.2)	11 (0.6)	1.7	0.54
***2nd repetition***						
P *(W.kg^*-1*^)*	6.4 (0.1)	6.3 (0.1)	8.3 (1)^b^	7.1 (0.3)^ab^	14.18	< 0.01
HR *(beats.min^*-1*^)*	103 (3)	101 (1)	105 (3)	103 (2)	1.2	0.13
%HRmax	54	55	54	54	1.7	0.23
RPE	11 (0.1)	11 (0.6)	11 (0.2)	11 (0.3)	1.9	0.35
***3rd repetition***						
P *(W.kg^*-1*^)*	6.1 (0.9)	6.3 (0.6)	8.2 (0.9)^b^	7.8 (0.2)^ab^	13.1	< 0.01
HR *(beats.min^*-1*^)*	103 (3)	101 (1)	105 (1)	104 (1)	3.1	0.33
%HRmax	53.5	54	54	55	2.1	0.21
RPE	11 (0.2)	12 (0.7)	12 (1.2)	11 (0.6)	1.1	0.54
***4th repetition***						
P *(W.kg^*-1*^)*	6.4 (0.3)	6.5 (0.5)	8.5 (1)^b^	7.7 (0.2)^ab^	11.7	< 0.01
HR *(beats.min^*-1*^)*	106 (3)	104 (2)	104 (3)	104 (1)	1.8	0.16
%HRmax	56	56	54	55	1.7	0.23
RPE	11 (0.4)	11 (0.6)	11 (0.2)	12 (0.2)	1.2	0.15
***5th repetition***						
P *(W.kg^*-1*^)*	6.5 (0.2)	6.4 (0.2)	8.6 (1)^b^	7.7 (0.5)^ab^	15.4	< 0.01
HR *(beats.min^*-1*^)*	105 (3)	103 (2)	103 (3)	103 (2)	1.9	0.18
%HRmax	54	55	54	54	1.6	0.22
RPE	11 (0.4)	11 (0.6)	11 (0.4)	11 (0.6)	1.1	0.12
***6th repetition***						
P *(W.kg^*-1*^)*	6.6 (0.1)	6.2 (0.5)	8.5 (1)^b^	7.5 (0.5)^ab^	21.41	<0.01
HR *(beats.min^*-1*^)*	104 (3)	102 (2)	104 (3)	102 (2)	1.9	0.18
%HRmax	54	55	54	54	1.6	0.22
RPE	11 (0.2)	11 (0.6)	11 (0.3)	11 (0.3)	1.1	0.12


*Values are mean ± SE (standard error). P, power developed at each repetition; 1, 2, 3, 4, 5, 6, number of repetition; HR, heart rate; %HRmax, percentage of maximal heart rate; RPE, rating of perceived exertion. ^*a*^Significant difference between groups (*p* < 0.01). ^*b*^Significant difference from pre-intervention values within an age group (*p* < 0.01).*

## Discussion

The first aim of the present study was to examine differences in the subjective sense of effort in relation to objective measures of intensities and associated physiological responses during maximal graded and SCE in sedentary young and older individuals. The second objective was to determine potential benefits of 6-weeks of supramaximal HIIT (i.e., SCE) intervention in such groups on the sense of effort as an intensity regulator. At the best of our knowledge, this study is the first to evaluate RPE during graded cycling test and during bouts of SCE as well as the effect of 6 weeks of SCE on RPE, and associated factors among young and older sedentary adults. The primary finding of the present work is the inconsistency found at baseline in perceived exertion between young and older participants at relative submaximal exercise intensities. Only the older group perceived the effort as being significantly higher as compared to the actual exercise intensity at VT1 and VT2 determined by objective methods. The 6-week SCE training program helped in normalizing perceived exertion values in the older group, bringing their RPE values determined at VT1 and VT2 to an adequate match of the actual exercise intensity classified by both %$\dot{V}$O_2_peak and %HRmax methods.

At baseline, the RPE values were significantly higher in older group compared to adults at the same relative exercise intensities corresponding to VT1 and to VT2 of the graded cycling test. In fact, in accordance to the Borg scale for ratings of perceived exertion (Borg, 1970) the older individuals perceived the two exercises stage (VT1 and VT2) as being *very hard* and *very very hard*. According to American College of Sport Medicine for intensity classification (Garber et al., 2011), the relative intensity determined in the present work using %HRmax and %$\dot{V}$O_2_peak methods correspond to *fairly light* for VT1 and to *somewhat hard* for VT2 which does not correspond adequately to the intensity perceived by RPE for the older group. At the contrary, young adult participants perceived VT1 and VT2 as *fairly light* and *hard* respectively corresponding with ratings observed by Alberton et al. (2016) that indicate values near to 16–17 when targeting a VT2 intensity for young women.

The discrepancy observed for RPE between our two groups may be explained by the age that has the potential to influence RPE as previously reported. Actually, some authors have reported that with aging many factors impair the cognitive functions among elders leading to alter their perceived exertion (Chodzko-Zazko and Moore, 1994; Boutcher, 2000). Accordingly, Grange et al. (2004) did not find any association between RPE and other physiological indicators (e.g., HR) during the course of a graded arm test to maximal exertion among inexperienced older group. At the contrary, Sidney and Shephard (1977) and Aminoff et al. (1996) reported that RPE is not impaired by aging and can be used as tool to control exercise intensity in healthy middle-aged and elderly individuals. For these authors perceived exertion is more affected by the physical fitness and the health status of the subject than by aging alone. In the present study, both our groups were sedentary and did not present previous exercise experiences, which could be revealing of an interaction effect of age and sedentary behavior on perceived exertion, given that only the older group presented altered sensory cues in the perception of effort. In such cases, to provide an accurate assessment and alternatively an appropriate individual exercise prescription, there is a need to combine RPE method with other commonly used methods of estimating relative intensity (e.g., HR) for older sedentary adults. Furthermore, introducing an acclimation period prior to any intervention should be considered among such a group to avoid any intensity misclassifications.

After 6 weeks of SCE, the RPE decreased significantly compared to baseline for the older group at VT1 and VT2 and was similar to those obtained for the young participants. Indeed, the two groups perceived the intensity as being *somewhat hard to hard*. This rate correspond adequately to what has been previously reported on the basis of %HRmax and %$\dot{V}$O_2_peak methods (Garber et al., 2011). The normalization of these values observed for older group at VT1 and VT2 seems to be mainly linked to significant improvements of older group’s ability to perceive effort since no significant associations were found with physiological indicators and no improvements in aerobic fitness were noted. These results lead us to suggest that training might have increased the subject’s abilities to perceive effort, which could be due to an improvement in memory or in the neuromuscular factors (Faulkner et al., 2008) given that no significant improvements in RPE values were seen throughout the intervention. Unfortunately, we did not evaluate neurophysiological adaptations nor memory capacities of our individuals. Therefore, considering these variables on further studies will be beneficial in determining the impact of such SCE model on cognitive function (e.g., attention, memory capacity) and on neurophysiological adaptations (e.g., blood flow, neurotransmitters) among older individuals and their relationship with RPE variations.

During the six bout of SCT, the RPE values were similar across groups at baseline and at post-intervention. In fact, both groups perceived the six bouts of SCT as being *fairly light* which might reveal an advantage for using very short bouts (6 s) of intense exercise. Indeed, an important consideration is that none of the participants withdrew from the intervention with a 100% compliance to the protocol throughout the 6 weeks of training. Moreover, participants displayed a constant relative power output during the six bouts of SCT indicative of a lack of actual fatigue at both baseline and during the post-intervention. This result may allow us to consider our model of SCE as a very appealing strategy for sedentary participants acknowledging the many physiological improvements seen at submaximal intensities in the post-intervention as compared to the baseline values in both groups and especially for the older one. Few studies have evaluated RPE during supramaximal HIIT-type intervention and no data is available for older sedentary individuals. However, it appears that both groups did not experience peripheral (i.e., muscular) and/or central (i.e., neural) fatigue during SCE; however, direct evidence to support this assumption is still lacking. In sum, we can conclude that the SCE regime accomplished in this study by repeating six “all-out” 6-s sprints on cycle ergometer favored a positive commitment among participants and seemed to be a desirable approach to adopt among an older sedentary population.

## Conclusion

To the best of our knowledge, this study is the first to examine RPE changes in response to 6 weeks of short bouts of supramaximal cycling intermittent exercise in young and older sedentary adults. Our analyses revealed that at baseline, the RPE values calculated at VT1 and VT2 for the older group did not correspond adequately to the relative intensity estimated by %HRmax and %$\dot{V}$O_2_peak methods. After the SCE intervention, RPE values were normalized and did not differ from the young adults. Careful attention should be paid on individual intensity assessment and monitoring to avoid any issues with negative consequences on exercise adherence. On the other hand, our study reveals that our SCE regime may be an appealing modality to introduce in older sedentary adults as a strategy aimed at improving exercise adherence, many submaximal physiological responses and therefore health status.

## Author Contributions

GJ contributed to the conception and design of the study and to the data collection. GJ and LM performed data analysis and interpretation, drafted the manuscript, and revised, read and approved the submitted version.

## Conflict of Interest Statement

The authors declare that the research was conducted in the absence of any commercial or financial relationships that could be construed as a potential conflict of interest.
